# Reversible Data Hiding in Encrypted Image Based on (7, 4) Hamming Code and UnitSmooth Detection

**DOI:** 10.3390/e23070790

**Published:** 2021-06-22

**Authors:** Lin Li, Chin-Chen Chang, Chia-Chen Lin

**Affiliations:** 1 Computer Engineering College, Ji Mei University, Xiamen 361021, China; jmulilin@jmu.edu.cn; 2 Department of Information Engineering and Computer Science, Feng Chia University, 100 Wenhwa Road, Seatwen, Taichung 40724, Taiwan; 3 Department of Computer Science and Information Engineering, National Chin-Yi University of Technology, Taichung 411030, Taiwan

**Keywords:** RDHEI, Hamming Code, MED, US (UnitSmooth), HUD-RDHEI, PSNR, ER

## Abstract

With the development of cloud storage and privacy protection, reversible data hiding in encrypted images (RDHEI) plays the dual role of privacy protection and secret information transmission. RDHEI has a good application prospect and practical value. The current RDHEI algorithms still have room for improvement in terms of hiding capacity, security and separability. Based on (7, 4) Hamming Code and our proposed prediction/ detection functions, this paper proposes a Hamming Code and UnitSmooth detection based RDHEI scheme, called HUD-RDHEI scheme for short. To prove our performance, two database sets—BOWS-2 and BOSSBase—have been used in the experiments, and peak signal to noise ratio (PSNR) and pure embedding rate (ER) are served as criteria to evaluate the performance on image quality and hiding capacity. Experimental results confirm that the average pure ER with our proposed scheme is up to 2.556 bpp and 2.530 bpp under BOSSBase and BOWS-2, respectively. At the same time, security and separability is guaranteed. Moreover, there are no incorrect extracted bits during data extraction phase and the visual quality of directly decrypted image is exactly the same as the cover image.

## 1. Introduction

Digital images are widely used in media, publishing, medicine, military, and other fields. Therefore, it is very important to protect the copyright and integrity of digital images. Because the image itself has the characteristics of large amount of data, high correlation and high redundancy between pixels, it cannot be used to encrypt the image with the common text encryption algorithm [1,2,3,4,5]. For above purposes, various technologies have been developed for images, such as image authentication [6,7,8] and watermarking [9,10]. As a branch of digital watermarking technology, data hiding is a critical technology to guarantee the security of confidential information. Data hiding can be implemented in many different ways to achieve the purpose of imperceptible embedding of secret data [11,12]. Depending on whether the receiver can fully recover the cover image, data hiding can be divided into two types: irreversible data hiding and reversible data hiding (RDH) [13,14].

**Figure 1:**
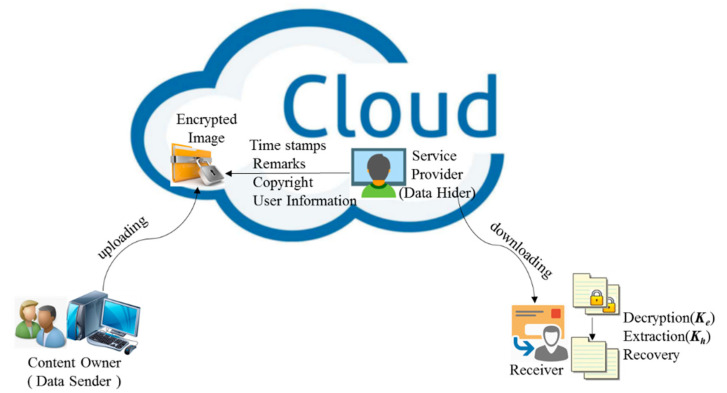
Sketch of RDHEI scheme.

With the growing maturity of cloud storage and cloud computing technology, data storage, image processing and other work has been transferred from local to cloud server. More and more data are uploaded to the cloud to be broadly shared, efficiently processed, stored centrally, or authenticated by a third party for the data source. In order to protect cloud stored data and user privacy, data will be encrypted before it is sent to the cloud server. For example, in the process of telemedicine, when the local hospital sends the patient’s medical image to the remote hospital through the network, the medical image needs to be encrypted before it is sent through communication channel to the cloud so that the patient’s privacy can be protected. At this time, the channel service provider may need to embed verification information in the transmitted data. As you can see from this example, the person who uploads the image, what we technically call the content owner, may want to upload encrypted images instead of plaintext images before adopting the functions offered by the cloud platforms. Encryption technology [15] can transfer the plaintext image into a meaningless image that is unrecognizable to the naked eye or even to powerful algorithms run in machines, which is technique we can apply in this scenario. Moreover, for the sake of management, some information such as owner information, timestamp, tagging, and source information of the encrypted media may need to be embedded into the encrypted image by service provider, also called as data hider. When needed, the user, more technically known here as the receiver can download the marked encrypted image to get the hiding message and original image with keys, as described in Figure 1. To achieve security and reversibility of the secret message, the RDH scheme is required to work on the encrypted image, which is called reversible data hiding in encrypted images (RDHEI) technique. RDHEI technique embeds the secret information into the encrypted image rather than the plaintext image, and the embedded secret information can be extracted correctly, along with the original plaintext image being recovered without loss. Therefore, RDHEI technique has the advantages of both RDH and encryption, realizing multimedia data privacy and copyright protection, content integrity authentication and ciphertext management, which is a research direction of intersection of data hiding and privacy protection.

**Figure 2:**
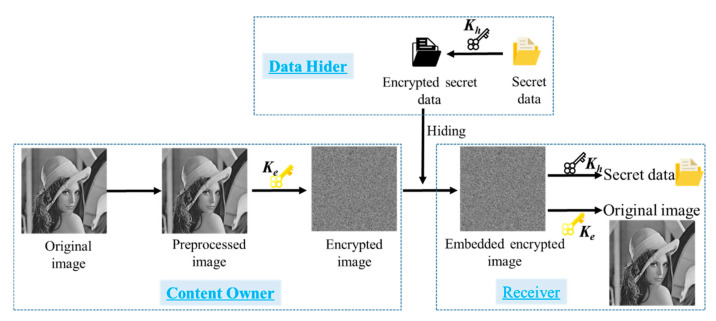
The overall process of RDHEI scheme.

As shown in Figure 2, there are usually three participants involved in RDHEI algorithm, which are content owner, data hider and receiver. In the RRBE scheme, the content owner will preprocess the original image to vacate hiding space, then the image is encrypted and sent to the data hider. Next, data hider embeds secrets into the encrypted images. Finally, the receiver can either use the hiding key *K_h_* to get secret data or use the encryption key *K_e_* to get the original image. Or, receiver can hold two keys to do both.

RDHEI algorithms can be divided into two categories, one vacates room after encryption and is called the VRAE algorithm [16,17,18] while the other reserves room before encryption and is called the RRBE algorithm [19,20,21]. Since VRAE algorithms perform the work of encrypting the image in the first place, the pixel correlation of the original plaintext image is completely destroyed, so the hiding capacity with VRAE algorithms is not high. By contrast, RRBE algorithms have a natural advantage in that they make use of the redundant space of the image to reserve as the hiding space before encryption, thus the hiding space of this algorithm is much larger than the former one. However, we believe the hiding capacity and the visual quality of the decrypted images still has the room to be improved while maintaining the good properties as the previously mentioned RRBE schemes. Therefore, a hybrid separable hiding strategy RDHEI is proposed in this paper.

The rest of the paper is arranged as follows. In the next section, knowledge of background information and related works, some closely related methods and the advantages of the proposed HUD-RDHEI scheme are present. In Section 3, the details of the proposed HUD-RDHEI scheme are described, including some new preprocessing methods designed by our research team are introduced. The experimental results and performance comparisons on ER and PSNRs with some state-of-the-art schemes are demonstrated in Section 4. Finally, a conclusion is given in Section 5.

## 2. Preliminaries

This section begins with some background information, then introduces and discusses some representative methods. At the end of this section, the highlights of the proposed HUD-RDHEI scheme and the key techniques used to overcome the current shortcomings of existing methods are given.

### 2.1. Backgound Information and Related Works

Data hiding is an interdisciplinary subject in the field of information security, covering cryptography, mathematics, computer vision and computer application technology. It is mainly to carry or hide secret information by using the redundancy in carrier data to achieve secure communication, copyright maintenance and other functions. It is one of the important research contents in the field of information security. Traditional information hiding mostly belongs to irreversible data hiding. In the process of embedding and extracting additional information, it will cause irreversible modification to the image and other carriers, and its application scenarios are relatively limited. Barton [22] first proposed the concept of reversible data hiding (RDH). So far, many RDH methods for plaintext images have been proposed [23,24,25]. The aim of these papers is to realize RDH method within images using interpolation-based scheme. Typically, the traditional RDH algorithms mainly use the following techniques, such as different expansion (DE) [26,27], histogram shifting (HS) [28,29], pixel value ordering (PVO) [30,31] and the modification of prediction errors [32,33]. All these methods take advantage of the spatial correlation and information redundancy between pixel values of the cover image to conceal secret message. These algorithms cannot directly work on the encrypted images due to correlations and redundancy cannot remain after the encryption processing. Thus RDHEI algorithms are proposed to solve data hiding in encrypted images, which are divided into two categories, VRAE and RRBE.

The idea of the VRAE method [16] was first introduced by Puech et al. in 2008. In their idea, the original image is encrypted with Advanced Encryption Standard (AES) and divided into blocks, then with the help of secret key *k*, the bit substitution-based method is used to replace a bit of a pixel for each block. The ER (embedding rate), which is the secret message bits carried by each pixel of an image, of Puech et al.’s scheme is 1/*n* bpp. With the analysis of the local standard deviation, the decryption and rebuilding of the original image can be performed. Unlike [16], in 2011 Zhang [17] used bit-wise cipher stream and XOR (EXCLUSIVE-OR) operation to encrypt an image. Then, the data hider segments the encrypted image into non-overlapping blocks. For each block, the encrypted pixels are randomly divided into two sets, S0 and S1. To embed a secret message, Zhang’s method flips the three least significant bits (LSBs) of each pixel of the encrypted image in set S0. At the receiver side, the decrypted image is divided into blocks, and one of the two sets is flipped to create new blocks. By measuring the fluctuation in two new blocks, data extraction and image restoration with an error rate can be realized. The error rate is directly affected by block size. When the block has 32 bits or more, most of the embedded bits of the cover image can be extracted correctly, and the original image can be restored successfully. Hereinto, the removing of embedded data takes place during the decryption process, so the data extracting and image recovering process are inseparable. In the next year, Zhang [18] designed a separable RDHEI method. First, using an encryption key, the content owner encrypts the original image. The data hider then adopts matrix operations to compress the LSBs of pixels in the encrypted image to make redundant room for the secret message. At the receiving end, depending on different keys, data extraction and image restoration can be performed separately. For an encrypted image that contains secret data, if the receiver has a data hiding key, s/he can extract the secret data with no information of the image. If the receiver has an encryption key, s/he can decrypt the received image and obtain an image similar to the original image without any secret data information. If both the data hiding key and the encryption key are owned by the receiver, they will extract the additional data and restore the original image completely error-free.

Due to the correlation of adjacent pixels that are damaged during the encryption processing, the hiding capacity of VRAE methods are relatively low in most cases and may encounter some errors in the procedure of data extraction and image recovery. In order to deal with such misjudgment problems, Ma et al. [19] proposed an RRBE method by reserving room before encryption. In their method, the original image is first divided into two parts: the smooth area and complex area. Then, standard RDH algorithms are applied to embed two or more LSB planes of the complex part into a smooth part to reserve room before encryption. After that, the preprocessed image is encrypted to generate the encrypted image. The vacated room of LSBs in complex parts can be applied for embedding a secret message. On the basis of the original algorithm, Mathew et al. [20] improved Ma et al.’s method in ER and PSNR, mainly adding the steps of dividing the original image into small blocks and classifying the small blocks into smooth blocks and complex blocks. In [21], Zhang et al. introduced a lossless, a reversible, and a combined data hiding scheme for ciphertext image encryption based on public-key cryptosystems with probabilistic and homomorphic properties. In the lossless scheme, the embedded data can be extracted directly from the encrypted image, and the embedding of the data has no effect on the decryption of the original plaintext image. In the reversible scheme, embedded data can be extracted, and the original image can be recovered from the directly decrypted image. Combined with these two schemes, the receiver can extract part of the embedded data before decryption, and extract another part after decryption, and restore the original plaintext image at the same time.

### 2.2. Some Closely Related Methods and the Advantages of the Proposed HUD-RDHEI Scheme

Unlike the algorithms mentioned above, in order to achieve the purpose of increasing embedding capacity, some recent RDHEI methods use an auxiliary bitmap to supplement the description of the pixel coordinates where the hidden information or the potentially misjudged information is located. Puteaux et al. introduced two methods in [34], CPE-HCRDH (high-capacity reversible data hiding approach with correction of prediction errors) and EPE-HCRDH (high-capacity reversible data hiding approach with embedded prediction errors), one using predictive error correction and the other using embedded predictive error. CPE-HCRDH corrects the prediction error by slightly modifying the pixel values. By sacrificing a little image quality, it can obtain a higher storage. In EPE-HCRDH, the pixel values are not modified, but the secret is not hidden in the block when its value of bitmap is equal to ‘1’. This method can obtain a high-quality image by sacrificing hiding capacity. After that, the image is bitwise encrypted. At the receiver end, the secret information can be reversibly extracted from the bitmaps. Based on a similar idea, Puyang et al. [35] introduced a two-MSB (MSB and the second MSB) prediction scheme. In the scheme, the correlation between adjacent pixels is well explored, and the reversibility, separability and error-free of data extraction are realized. At the same time, the aim of increasing the hiding capacity is achieved. However, the scheme proposed by Yi et al. in [36] is different from [35]. In [36], the PBTL-DE (parametric binary tree labeling scheme data embedding) method is applied to the encryption domain by utilizing spatial redundancy, and a reversible data hiding method (PBTL-RDHEI) based on PBTL is proposed. Separately, the recovery of the original image and the extraction of the secret data can be realized in a lossless manner. When the block size is set to 2 × 2 and 3 × 3, the average ER can reach 1.752 bpp and 2.003 bpp, respectively. Recently, Chen et al. proposed a scheme in [37], in which a joint lossless compression scheme is realized to vacate embedding room. Image recovery can be realized from encrypted images with only the encryption key directly, and secret data extraction can be achieved with only the data hiding key.

The proposed scheme belongs to the RRBE RDHDI method, and it is hybrid, separable and completely reversible. The highlights of the proposed HUD-RDHEI scheme are as follows:

(1) The (7, 4) Hamming Code is adopted to encode the LSBs of the original image to partial non-LSBs of pixels in an image to vacate room for the data hider so that the secret message can be concealed safely.
(2) A novel detection function US is defined and used to guarantee the hiding capacity is highly improved.
(3) A flipping-MSB operation combined with the MED prediction method to record the modified position of the non-LSBs is applied to achieve the complete recovery without any error.
(4) Secret information is encrypted before being hidden, at the same time, the auxiliary information used for recovery is also encrypted, which increases the security.
(5) The proposed HUD-RDHEI scheme uses stream encryption to avoid the information leakage and plaintext attack caused by edge effect and other unsafe risks.
(6) The proposed HUD-RDHEI scheme is separable. It enables the scheme to be applied in a wider range of scenarios.

## 3. The Proposed HUD-RDHEI Scheme

**Figure 3:**
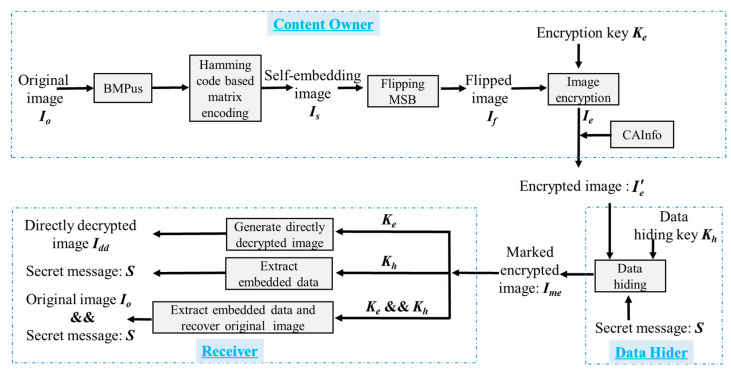
The framework of the proposed HUD-RDHEI scheme.

The framework of our proposed RDHEI scheme is depicted in Figure 3. It is noted that there are three phases: encrypted image generation, data hiding, and data extraction/image recovery, and two keys are conducted at different phases. At the beginning, the content owner uses an encryption key *K_e_* to encrypt image, with the vacating room for hiding at the same time. Then, data hider uses a hiding key *K_h_* to hide the secret data in the vacated room. At the receiver side, s/he can obtain different images based on which key(s) s/he holds: (a) obtaining a directly decrypted image if s/he only has an encryption key *K_e_*, (b) obtaining hidden secret data if s/he only has a hiding key *K_h_*, and (c) obtaining the original image and the hidden secret data as long as s/he holds two keys: *K_e_* and *K_h_*.

To improve the hiding capacity, we first designed the US (UnitSmooth) function. Then, a self-embedding method based on (7, 4) Hamming Code [38,39,40] and our proposed flipping-MSB method is proposed. For the content owner, the proposed UnitSmooth bitmap (BMPus) according to US function is recorded first. Then, a (7, 4) Hamming Code is used to hide the LSB bits to partial non-LSB bits of the pixels. Through these two steps, two rooms for hiding information are reserved. Then, the image will be encrypted and sent to the data hider, with CAInfo (compressed auxiliary information) embedded. At the data hider side, the LSBs and the 6 bits of UnitSmooth pixels of the received encrypted image are substituted with binary bits of the secret message, which has been scrambled with the data hiding key *K_h_*. On the receiver end, processing is different on the basis of the keys held by the receiver end and the role the receiver plays. If the receiver is a data hider and only holds data hiding key *K_h_*, s/he can extract the hidden message without knowing any information of the original image. If the receiver is an image owner and s/he only holds the encryption key *K_e_*, s/he can decrypt the marked encrypted image to generate a directly decrypted image with visual quality of PSNR tending to +∞. This can also be regarded as the proof of the separability of the algorithm. If the receiver holds both the data hiding key *K_h_* and the encryption key *K_e_*, s/he would not only perform the data extraction but also the error-free image recovery.

The following subsections describe US function, data hiding method based on (7, 4) Hamming Code, MED prediction and our defined compressed auxiliary information (CAInfo) in detail.

### 3.1. US Function and BMPus

Here we define and utilize the unit smoothness of the cells to improve the hiding capacity for the data hider. A bitmap of unit smoothness (BMPus) of all the cells of the image will be calculated and recorded for complete recovery when needed. The details are shown as follows: when a grayscale image *I_o_* of size 512 × 512 is given, according to the raster scan sequence, except the first line and first column, we consider every three consecutive pixels $Pixel_{k}^{1}$, $Pixel_{k}^{2}$, and $Pixel_{k}^{3}$ as a non-overlapping cell. Thus, there will be (M−1)×⌊(N−1)/3⌋ cells in the image *I_o_*, each of which is called *Cell_k_*, where k∈[1,(M−1)×⌊(N−1)/3⌋]. The conversion between the cell number and the coordinate (*i*, *j*) of a pixel in Io is formulated as Equation (1):

(1) $$k=\left\lfloor \frac{\left(i-2)\times 510+(j+1\right)}{3}\right\rfloor ,$$

where *i* ∈ [2, 512], *j* ∈ [2, 511] and ⌊⋅⌋ rounds down a real number to the nearest integer that is smaller than it.

**Figure 4:**
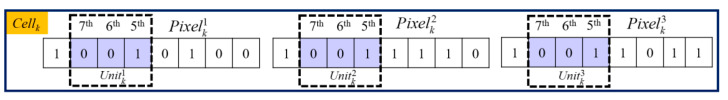
The cell structure of *Cell_k_*.

**Figure 5:**
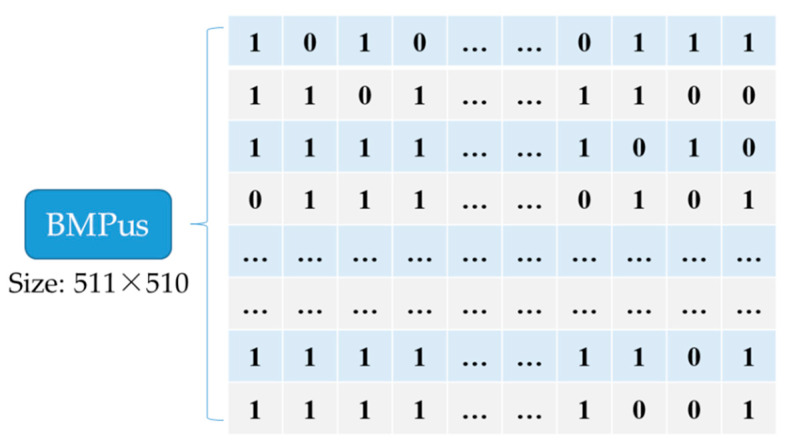
A bitmap of unit smoothness (BMPus) example.

Then we define a utility function called the US function to measure the smoothness of each cell based on the cell structure depicted in Figure 4. For each pixel, its 7th to 5th bits are selected as a unit. Three determined units are compared with each other to generate the US value according to Equation (2) for a given *Cell_k_*:

(2) $$\mathrm{US}(Cell_{k})=\left\{ \begin{array}{l} \;1,\;if\;bitget(Pixel_{k}^{1},7,-1,5)=bitget(Pixel_{k}^{2},7,-1,5)=bitget(Pixel_{k}^{3},7,-1,5)\\ \;0,\;else, \end{array}\right.$$

where the US function is used to determine whether a cell is smooth or not, and $bitget(Pixel_{k}^{i},7,-1,5)$ means to fetch the 7th to 5th bits of $Pixel_{k}^{i}$ to form a 3-bits binary unit. As described in Figure 4, $Unit_{k}^{1}$=$\;bitget(Pixel_{k}^{i},7,-1,5)\;$ = ‘001’, $Unit_{k}^{2}$ = ‘001’, $Unit_{k}^{3}$ = ‘001’, and the conclusion can be drawn that $Unit_{k}^{1}$ = $Unit_{k}^{2}$ = $Unit_{k}^{3}$. According to the definition of US in Equation (1), the function result of $\mathrm{US}(Cell_{k})$ is ‘1’ in this case. On this occasion, $Unit_{k}^{2}$ and $Unit_{k}^{3}$ can be cleared and used to embed a secret message without any worry about not being able to recover, because $Unit_{k}^{1}$ has saved their values. A BMPus is used to indicate if the corresponding unit is smooth or not is generated, as demonstrated in Figure 5, where ‘1’ represents $\mathrm{US}(Cell_{k})=1$. 

Such $Cell_{k}$ is called a USC, which means the *k*th UnitSmooth Cell in an image by using raster scan sequence to generate the serial number *k*. USCs can be used to hide the secret message. By contrast, ‘0’ in the BMPus represents $\mathrm{US}(Cell_{k})=0$, and this cell is un-embeddable. For an image with a size of 512 × 512, the bitmap will have 86,870 bits, which is calculated as 511×⌊511/3⌋ since the first column and first row are reference pixels. The content of the BMPus will be embedded orderly into the vacated LSBs of the encrypted image for the receiver can extract the secret information according to this map when needed.

### 3.2. Self-Embedding Method Based on the (7, 4) Hamming Code

**Figure 6:**
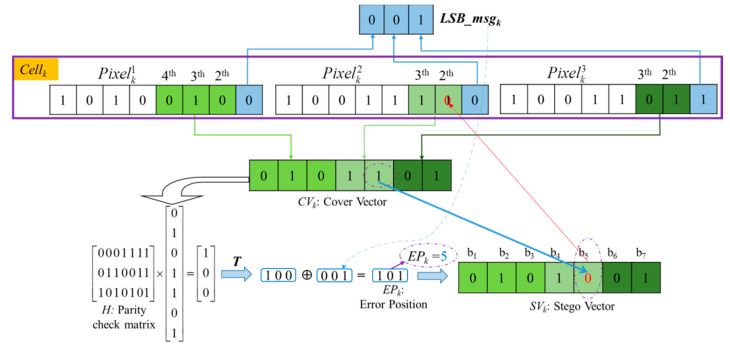
Self-embed *LSBmsg_k_* into *CW_k_* to obtain $C{W^{\prime }}_{k}$.

Hamming Codes [38] were introduced in 1950 by Richard W. Hamming as a way of automatically correcting errors. Generally, the basic idea of Hamming Codes is the concept of parity check [39]. In 2008, one of our authors applied a (7, 4) Hamming Code to design a data hiding method [41]. Inspired by Chang et al.’s concept in [41], here we use a (7, 4) Hamming Code to self-embed the LSBs of each pixel in a *Cell_k_* into the specific non-LSB bits as shown in Figure 6, and then the LSB of three pixels in a *Cell_k_* is created as the second room for concealing secret data.

*Step 1.* Concatenate all LSBs of three pixels in *Cell_k_* to form a 3-bits binary code *LSBmsg_k_* and its decimal value is ranged of [0, 7].

*Step 2.* Combine the 4th to 2nd bits of $Pixel_{k}^{3}$, the 3^rd^ to 2^nd^ bits of $Pixel_{k}^{2}$ and the 3rd to 2nd bits of $Pixel_{k}^{1}$ to form a 7-bits codeword called *CW_k_*. Then, let *X* = *CW_k_* and obtain *Y* according to Equation (3):

(3) $$Y=f(X)=H\times X^{T}=\left[\begin{array}{ccccccc} 0 & 0 & 0 & 1 & 1 & 1 & 1\\ 0 & 1 & 1 & 0 & 0 & 1 & 1\\ 1 & 0 & 1 & 0 & 1 & 0 & 1 \end{array}\right]\times X^{T},$$

where *H* is called a parity check matrix.

*Step 3.* Obtain error position (*EP_k_*) after encoding *LSBmsg_k_* into *CW_k_* by Equation (4). Finally, the bit value located at *EP_k_* is flipped in *CW_k_* to generate a stego codeword $C{W^{\prime }}_{k}$:

(4) $$EP=Y^{T}⊕LSBmsg_{k}.$$

### 3.3. The Proposed Flipping-MSB Method

**Figure 7:**
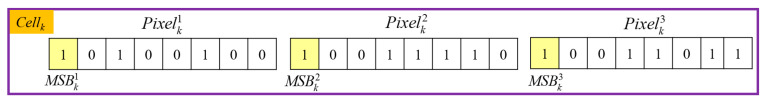
Three MSBs of *Cell_k_*.

To make sure the original 7-bits codeword *CW_k_* can be completely restored at the receiver side, in our proposed scheme, the error position *EP_k_* must be recorded in advance. Here, we designed a flipping-MSB method to record *EP_k_*. The core concept of the flipping-MSB method is based on the MSB of three pixels in a *Cell_k_*. To be specific $MSB_{k}^{1}$, $MSB_{k}^{2}$ and $MSB_{k}^{3}$ are used to indicate the MSB of three pixels in *Cell_k_*, respectively, as denoted in Figure 7.

With using three MSB values $MSB_{k}^{1}$, $MSB_{k}^{2}$ and $MSB_{k}^{3}$, there are eight combinations to indicate the error position *EP_k_* ranged in [0, 7] shown in Table 1. After the pixels in all the MSBs of cells have been flipped according to their *EP_k_*, the flipped image is produced and named as *I_f_*.

### 3.4. MED Prediction and Our Defined Compressed Auxiliary Information (CAInfo)

**Figure 8:**
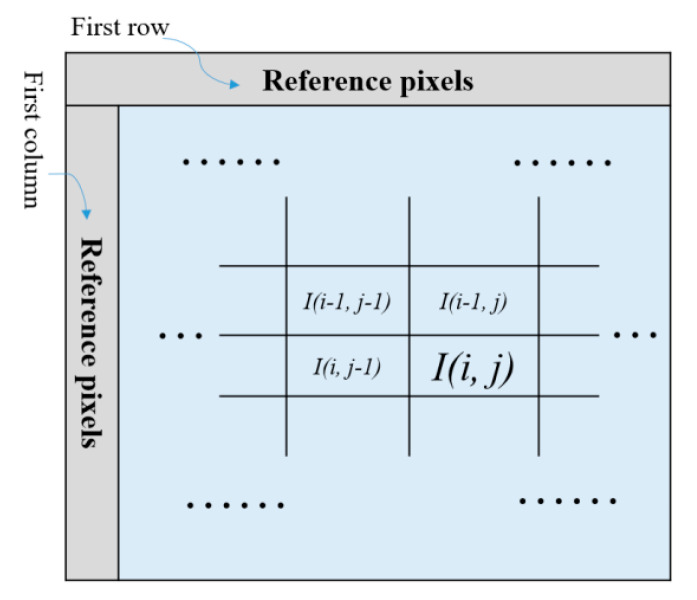
Example of reference pixels, the current pixel, and its three neighboring pixels.

In the previous subsections, we mentioned the MSB of three pixels in a *Cell_k_* may be flipped to record *EP_k_*. To make sure the correct MSB of three pixels in a *Cell_k_* can be obtained later, here Weinberger et al.’s median-edge detector (MED) [41] is adopted. For an original image *I* sized *M* × *N* pixels, pixels located in the first row and first column are served as the reference pixels, according to Equation (5). For a current pixel *I* (*i*, *j*), its predicted value is then derived based on its three adjacent pixels, which are located at the top left, top, and left of the sample, as shown in Figure 8.

The predicted value generated by MED is used to indicate whether the received MSBs have been flipped, based on our assumption: *D_o-med_* should be smaller than *D_o-fmed_* in general, where *D_o-med_* is the absolute value of the difference value between the predicted pixel value and the original pixel value, and *D_o-fmed_* is the absolute value of the difference between the predicted pixel value and the original pixel value carrying the flipped MSB. To prove our assumption, the comparisons are listed in Table 2.

(5) $$I{\left(i,j\right)}_{MED}=\left\{ \begin{array}{l} \min(I(i-1,j-1),I(i-1,j)),\;ifI(i,j-1)\geq \max(I(i-1,j-1),I(i-1,j));\\ \max(I(i-1,j-1),I(i-1,j))\begin{array}{c} ,ifI(i,j-1)\leq \min(I(i-1,j-1),I(i-1,j)) \end{array};\;\\ I(i-1,j-1)+I(i-1,j)-I(i,j-1),\;otherwise. \end{array}\right.$$

From Table 2, we can see only 111 pixels for ‘*Lena*’, whose *D_o-fmed_* is less than *D_o-med_*. Even in the worst case such as ‘*Baboon*’, there are only 3630 pixels where *D_o-fmed_* is less than *D_o-med_*. In other words, as long as the coordinates of all exceptional pixels and their MSB values are recorded, the abnormal situation can be detected in advance, thus ensuring the complete recovery of the original image. The coordinates of all exceptional pixels and their MSB values, named as EXCmsb, concatenated with BMPus will be compressed with the Lossless JBIG2 algorithm [42] to further shrink its size, and the compressed result is called compressed auxiliary information (CAInfo) with length of *l*, which will be embedded into the LSBs of the first *l* pixels of the encrypted image later.

Based on the four designed functions and methods described in previous sections, our proposed HUD-RDHEI scheme can easily achieve its two objectives: high capacity and error-free at the recovery phase. Following existing RDHEI schemes, three participant roles, namely content-owner, data-hider, and receiver, are included in our proposed scheme.

**Figure 9:**
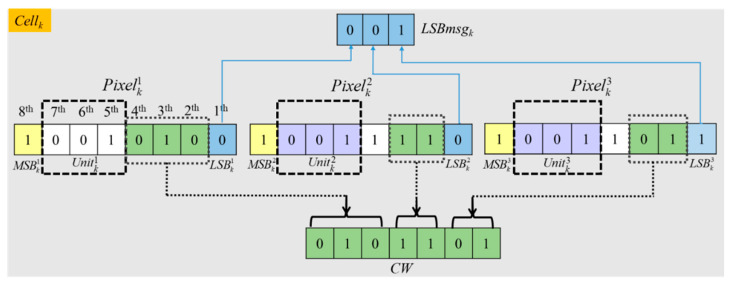
Hiding strategy for a cell.

The primary hiding strategy adopted in our proposed scheme is shown in Figure 9. Initially, an original image is divided into non-overlapping cells, and each cell contains three neighboring pixels. For an image with size of *M* × *N*, with leaving the first row and the first column intact, there will be (M−1)×⌊(N−1)/3⌋ cells. For a cell *Cell_k_*, two parts of embedding rooms are then generated to carry a secret message and CAInfo, which guarantees the original image can be restored without error. The first part of vacated room is marked in purple and the second one is marked in blue in Figure 9.

Here every cell has three units $Unit_{k}^{1}$, $Unit_{k}^{2}$, and $Unit_{k}^{3}$, and a unit consists of the 5–7th bits of a pixel, as shown in Figure 9. The first part of vacated room is 6-bits, which are derived from the latter two units $Unit_{k}^{2}$ and $Unit_{k}^{3}$ in a cell when three units in the cell have the same values. That is $\mathrm{US}(Cell_{k})=1$ and colored in purple Figure 9. The second part of vacated room is 3-bits, which is the collection of the LSBs of three pixels in *Cell_k_*. It will be used to hide CAInfo and a part of secret message. To save the original value of the threes LSBs in *Cell_k_*, which are called *LSBmsg_k_*, the self-embedding method based on the (7, 4) Hamming Code described in Section 3.2 are applied. During the procedure, the combination of the 4th–2nd bits of the first pixel and the 3rd–2nd bits of the second and third pixels are combined and named as *CW* then used to carry *LSBmsg_k_*.

With our designed two vacated room strategies, a large amount space for secret data and the required auxiliary information are successfully created, generation of marked encrypted image, completely restoring the original image and extracting secret information can be achieved as well when needed. The detailed operations in three phases are demonstrated in the following subsections.

### 3.5. Encrypted Image Generation

Given an original grayscale image *I_o_* of width *M* and height *N*, on the content owner side, there are 7 Steps to generate the corresponding encrypted image carrying the auxiliary information CAInfo:

*Step 1:* Except pixels located in the first line and first column, group three neighboring pixels in the original image Io into Cells. Compute the US function and generate a BMPus according to Section 3.1.

*Step 2:* Concatenate all the LSBs of the three pixels in *Cell_k_* to form a 3-bits binary string *LSBmsg_k_*, then encode *LSBmsg_k_* into *CW_k_* acquired from the non-LSB bits to obtain $C{W^{\prime }}_{k}$ according to the self-embedding method based on the (7, 4) Hamming Code discussed in Section 3.2. Thus, the self-embedding image *I_s_* and error position *EP_k_* are obtained.

*Step 3:* Flip $MSB_{k}^{i}$ on the basis of our proposed flipping-MSB method presented in Table 1 to record *EP_k_*. Thus, the flipped image *I_f_* is generated.

*Step 4:* Produce the MED prediction value of all pixels except the first column and the first row (the first column and the first row will be used as reference pixel values and kept unchanged). The two groups of difference values are generated, namely, *D_s-med_*, which is the difference of pixel value and MED prediction, and *D_sf-med_* that is the difference of pixel value with a flipped MSB and MED prediction. Find out all the pixels that $\;D_{s-med}\geq D_{sf-med}$. If they are found, the coordinates and MSB values of the pixels will be recorded, which are named EXCmsb as mentioned in Section 3.4.

*Step 5:* Use the encryption key *K_e_* to perform a bitwise encryption on the flipped image *I_f_* to generate the encrypted image *I_e_*. The encryption algorithm is rendered as Equations (6)–(8):

(6) $$I_{f}(i,\;j,\;u)=\left\lfloor I_{f}(i,\;j)/2^{u-1}\right\rfloor \;\mathrm{mod}\;2,$$

(7) $$I_{f}(i,\;j)=\sum_{u=1}^{8}\left(I_{f}(i,\;j,\;u)\times 2^{u-1}\right),$$

(8) $$I_{e}(i,\;j,\;u)=I_{f}(i,\;j,\;u)\;⊕\;r(i,\;j,\;u),$$

where u∈[1, 8], i, j∈[0, 255] and *r*(*i*, *j*, *u*) is a binary bit value ‘1’ or ‘0’ produced in sequence according to a bit stream {0, 1}, which is determined by the encryption key. And $I_{f}(i,\;j,\;u)$ is a bit of the pixel $I_{f}(i,\;j)$ of the flipped image If, where $I_{f}(i,\;j,\;1)$ is the LSB of $I_{f}(i,\;j)$, and $I_{f}(i,\;j,\;8)$ is the MSB.

*Step 6:* Generate the CAInfo with the Lossless JBIG2 algorithm [33] to further shrink its size of CAInfo = BMPus || EXCmsb, where ‘||’ means the concatenation of the two parts of bit stream and EXCmsb.

*Step 7:* Embed CAInfo into the first *l* LSBs of the encrypted image *I_e_* to form ${I^{\prime }}_{e}$, which will be sent to the data hider.

**Figure 10:**
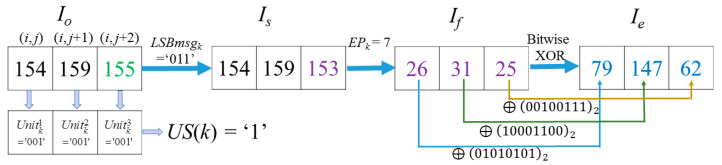
Example-1 of the processing procedure and encryption of *Cell_k_*.

*Example-1***:** For image ‘*Lena*’, when *k* = 48,367, *i* = 286 and *j* = 260 according to Equation (1). There are three pixels in the *Cell_k_* as $Pixel_{k}^{1}=I_{o}(i,\;j)=154,$ $\;Pixel_{k}^{2}=I_{o}(i,\;j+1)=159,$ $\;Pixel_{k}^{3}=I_{o}(i,\;j+2)=155.$ The encryption procedure of the three pixel values in *Cell_k_* are demonstrated in Figure 10.

In this case, the 7th, 6th, and 5th bit of *I_o_*(*i*, *j*), *I_o_*(*i*, *j* + 1) and *I_o_*(*i*, *j* + 2) constitutes $Unit_{k}^{1}$$Unit_{k}^{2}$, and $Unit_{k}^{3}$, respectively. Because the three units are equal, the corresponding US(*Cell_k_*) = ‘1’. This means the last two units $Unit_{k}^{2}$ and $Unit_{k}^{3}$ of *Cell_k_* can be applied to hide secret data. The LSBs of the three pixels is ‘0’, ‘1’, and ‘1’; therefore, *LSBmsg_k_* = ‘011’. *CW* = ‘1011101’ is formed by those bits at the specified location, which are the 4th, 3rd, and 2nd bit of $Pixel_{k}^{1}$, 3rd and 2nd bit of $Pixel_{k}^{2}$, and 3rd, 2nd bit of $Pixel_{k}^{3}$. After conducting data hiding based on the (7, 4) Hamming Code, $P_{{\mathrm{EP}}_{k}}$ = b_7_, which means the 2nd bit of the $Pixel_{k}^{3}$ value should be flipped. After the flipping operation, *CW* is encoded into $CW^{\prime }$ = ‘1011100’. Thus, *I_s_*(*i*, *j*) = *I_o_*(*i*, *j*) = 154, $I_{s}(i,j+1)=I_{o}(i,j+1)=159,\;$*I_s_*(*i*, *j* + 2) = 153. Note that only the last bit of the three pixels in *Cell_k_* is changed. Due to *EP_k_* = 7, according to Equation (3), flipping $MSB_{k}^{i}$(i∈[1, 3]) of the three pixels in *I_s_* is selected according to flipping rules listed in Table 1. Thus, the flipped pixel values *I_f_*(*i*, *j*) = 26, *I_f_*(*i*, *j* + 1) = 31, *I_f_*(*i*, *j* + 2) = 25 are obtained. *I_med_*(*i*, *j*) = 156, *I_med_*(*i*, *j* + 1) = 154, *I_med_*(*i*, *j*) = 159. Thus, |154−156|<|26−156|, which is the comparison result of the two difference values between the MED prediction value *I_med_*(*i*, *j*) and the values before and after flipping the MSB of *I_s_*(*i*, *j*). Obviously, it corresponds to the general case, so their coordinates and MSB values will not be recorded as mentioned in Section 3.4. The other two pixels in *Cell_k_* are also the general case. Finally, after the XOR operation, the three encrypted pixels in *Cell_k_* are derived as *I_e_*(*i*, *j*) = 79, *I_e_*(*i*, *j* + 1) = 147 and *I_e_*(*i*, *j* + 2) = 62, as shown in Figure 10.

**Figure 11:**
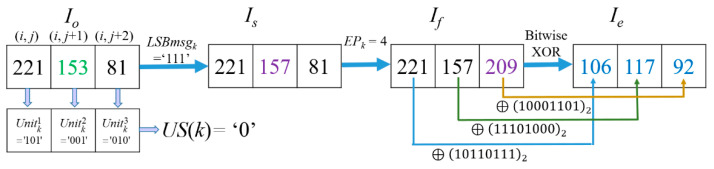
Example-2 of the processing procedure and encryption of *Cell_k_*.

*Example-2:* For image ‘*Lena*‘, when *k* = 65,984, *i* = 390, *j* = 71 according to Equation (1), $Pixel_{k}^{1}=I_{o}(i,\;j)=\;221$, $Pixel_{k}^{2}=I_{o}(i,\;j+1)=153$, $Pixel_{k}^{3}=I_{o}(i,\;j+2)=\;81.$ The encryption processing procedure of the pixel values in the *Cell_k_* is demonstrated in Figure 11.

It is noted that US (*Cell_k_*) = 0 in this case because their $Unit_{k}^{1}$ = ‘101’, $Unit_{k}^{2}$ = ‘001’, and $Unit_{k}^{3}$ = ‘010’ are not equal. Thus, the last two units $Unit_{k}^{2}$ and $Unit_{k}^{3}$ of *Cell_k_* cannot be used to hide any secret data. Here, *LSBmsg_k_* = ‘111’. After the hiding process based on the (7, 4) Hamming Code, the *CW* is changed into $CW^{\prime }$ = ’1101000’ by flipping the bit on *b_4_* because *EP_k_* = 4. Thus $I_{s}(i,j)=I_{o}(i,j)=\;221,\;$$I_{s}(i,j+1)=I_{o}(i,j+1)$ = 153, $\;I_{s}(i,j+2)=\;81$. Flip $MSB_{k}^{i}$ of $I_{s}(i,j+2)\;$ in *Cell_k_* due to *EP_k_* = 4 according to flipping rules listed in Table 1. Among them, *I_s_*(*i*, *j* + 2) = *I_s_*(390, 73) = 81, *I_med_* (390, 73) = 153, the value with the flipped MSB of *I_s_*(*i*, *j* + 2) is 209, which shows |81−153|>|209−153|, thus the coordinate and the MSB value of *I_s_*(390, 73) will be formed as a 17-bits binary string for saving to CAInfo. After the flipping operation, the flipped image *I_f_* (*i*, *j*) is obtained. Finally, we conduct a bitwise XOR on every bit of the three pixels to get the three encrypted pixel values as $I_{e}(i,\;j)=\;106,$$I_{e}(i,\;j+1)=117,$ and $\;I_{e}(i,\;j+2)=\;92$.

### 3.6. Data Embedding in the Encrypted Image

On the data hider side, the LSBs of all the pixels of the encrypted image *I_e_* can be applied to embed a secret message. In addition, it is particularly noteworthy that more secret data will be hidden in the last two Units of USCs. Taking the grayscale image ‘*Lena*’ with size of 512 × 512 as an example, the number of USCs is 69.6 percent of the total number of cells. Then, to avoid the potential information theft risk, the data hiding key *K_h_* is used to scramble the secret message *S*, which randomizes the order of bits in binary numbers. Furthermore, the scrambled secret message $S^{\prime }$ is carried out, bits of which will be substitution bits to replace the LSBs and next the last two Units of USCs to obtain the marked encrypted image *I_me_*.

**Figure 12:**
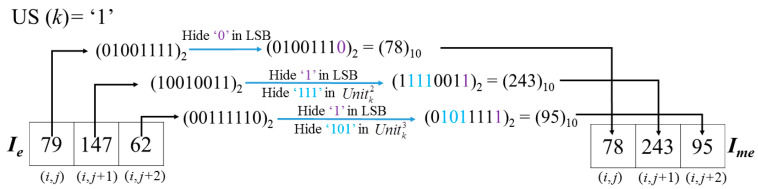
Example of embedding of secret message.

*Example-3:* For grayscale image ‘*Lena*’, when *k* is 48,367, *i* = 286, *j* = 260. (Here ${I^{\prime }}_{e}(i,j)$ = $I_{e}(i,j)$ because the CAInfo of ‘*Lena*’only needs *l* = 888 bits to hide and will not influence the pixels when *k* > 296 (=888/3)). After the encryption operation with *K_e_*, $Cell_{k}^{e^{\prime }}$ = (79, 147, 62). Based on the BMPus value uncompressed from CAInfo, the hiding scrambled secret message procedure is listed as follows in Figure 12.

**Figure 13:**
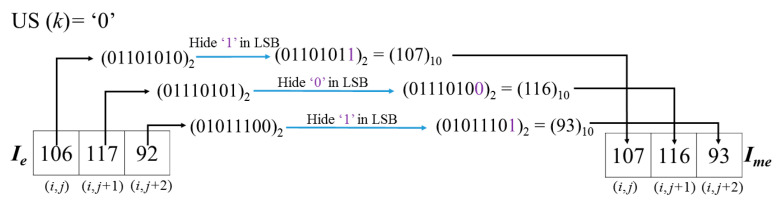
Example of embedding of secret message.

As one can see, the US(*Cell_k_*) value will first be judged to determine whether the last two units of $Cell_{k}^{e^{\prime }}$ are embeddable. Due to US(*Cell_k_*) = 1 in this example, we can embed 9 bits in the three pixels of $Cell_{k}^{e^{\prime }}$ in *I_e_* here. Thus, the embedding ratio (ER) of this cell is 9/3 = 3 bpp. This procedure will be processed for all pixels in the embeddable area of the image. By contrast, if US(*Cell_k_*) = 0 as shown in Figure 13, the last two units of $Cell_{k}^{e^{\prime }}$ are un-embeddable. In this case, only three bits can be carried in a Cell.

### 3.7. Data Extraction and Image Recovery

In this section, the details of data extracting and image recovering process of the proposed HUD-RDHEI scheme will be introduced.

#### 3.7.1. Data Extraction with Only a Data Hiding Key

If the receiver holds only the data hiding key, they can take out the first *l* LSB bits that represents the content of CAInfo by raster scan order pixels of marked encrypted image. Based on the derived CAInfo, the extracted BMPus can be used to identify which cells are embeddable. For the embeddable cells, six secret bits can be extracted from the last two units. As for the extracted *l* + 1 bits, they are treated as the second part of the secret message. Combine the first secret data set and the second secret data set to be the final encrypted secret message. With the data hiding key, the encrypted secret message can be decrypted, and the final restored secret message is obtained. Note that the extracted decrypted secret data is lossless, and the extraction process is executed without any information about the content of the original image.

#### 3.7.2. Image Decryption with Only the Data Encryption Key

On this occasion, the receiver has the encryption key *K_e_* only; s/he can obtain the directly decrypted image optimally close to the original image with PSNR of +∞:

*Step 1.* Take the first *l* bits, bit by bit, out from the LSB bits of the marked encrypted image *I_me_* to form CAInfo.

*Step 2.* Generate the random {0, 1} bit stream using *K_e_*, then execute the bitwise XOR operation with the received image bit by bit.

*Step 3.* Recover the latter two 3-bits units of USCs using the value of the first 3-bits unit in a cell of *I_me_*. Now the Flipped image *I_f_* is obtained.

*Step 4.* Flip back the pixel value, pixel by pixel, according to which condition it meets. If the corresponding coordinate is not recorded in EXCmsb, it means the case is normal. Compare the two difference values, one difference value is between the MED prediction value and the MSB unflipped pixel value, the other difference value is between the MED prediction value and the MSB flipped pixel value. If the former is less than the latter, it is a normal case and no flipping operation is required; otherwise, MSB must be flipped back. If the pixel’s coordinate is found in EXCmsb, it is determined as an abnormal case, and the following bit is its original MSB.

*Step 5.* Compare the MSB extracted from EXCmsb and the MSB extracted from the flipped image with each other. If they are the same, it means no flipping has been conducted during the data hiding phase; otherwise, it means that flipping has been conducted. Extract the flip information of MSBs in a cell to get the value of *EP_k_* according to Table 1. The bit value on position *EP_k_* is where *CW* is different from $CW^{\prime }$. Flip back that bit then the original *CW* value is obtained.

*Step 6***.** Substitute the *X* value with $CW^{\prime }$ and execute the computation of Equation (3), then the original *LSBmsg_k_* value can be available. Take the values of the three bits of *LSBmsg_k_* back to the LSBs of the three pixels in the cell, the original pixel values are carried out finally.

Hereinto, *I_med_*(*i*, *j*) can be derived pixel by pixel during the decryption process from the first pixel located in the first row and first column. This is because pixels located at the first row and the first column remain unchanged and serve as reference pixels. Therefore, prediction values for the rest of the pixels can be correctly calculated with the assistance of these reference pixels.

**Figure 14:**
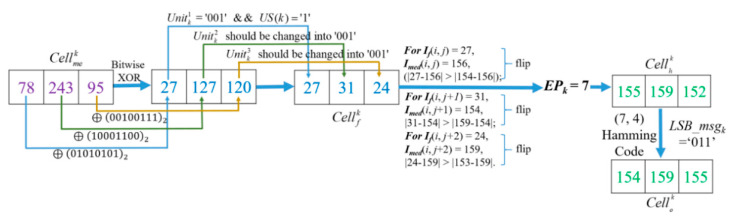
Example of image decryption procedure with *K_e_*.

*Example-4:* Here, follow the encrypted and hidden result demonstrated in Figure 12, and the corresponding decryption procedure with encryption key *K_e_* is demonstrated in Figure 14.

After decrypting all the pixels of *I_me_* with *K_e_*, the values of three decrypted pixels of $Cell_{k}^{me}$ = [27, 127, 120] will be found. Note that, during the decryption procedure, the bit strings that are executed on pixels with bitwise XOR are as same as the encryption procedure due to the same key *K_e_*. The binary value of 27 is ‘00011011’, thus according to method described in Figure 4, $Unit_{k}^{1}=’001’$ can be obtained. US(*Cell_k_*) value ‘1’ can also be obtained from the first *l* LSB bits of ${I^{\prime }}_{e}$ that symbolize the CAInfo of an image *I_o_*. Here, the obtained value ‘1’ means the last two units are determined as USC, and they are embedded with a secret message and changed to recover their original bits, and $Unit_{k}^{2}$ and $\;Unit_{k}^{3}\;$ shall be modified as ‘001’ to get $Cell_{k}^{f}$ = [27, 31, 24 ]. Since the coordinates of these three pixels have not been recorded in EXCmsb, all of them are judged as the normal case. However, for the normal case, the difference between the MED prediction value and the MSB unflipped pixel value should be less than the difference between the MED prediction value and the MSB flipped pixel value. Unfortunately, it is not held in three pixels. It indicates that the MSBs were flipped in the hiding stage. Therefore, for recovery, the MSBs of three pixels must be flipped back to get [155, 159, 152]. Because three pixels have been flipped, it is concluded that *EP_k_* = 7 and the 2nd bit of  was modified during the data embedding procedure according to the rules listed in Table 1. Finally, the original $Cell_{k}^{o}$ [154, 159, 155] can be obtained with the derived *LSBmsg_k_* = ’011’ from calculation of Equation (3) and flipping the 2nd bit of $Pixel_{k}^{3}$.

**Figure 15:**
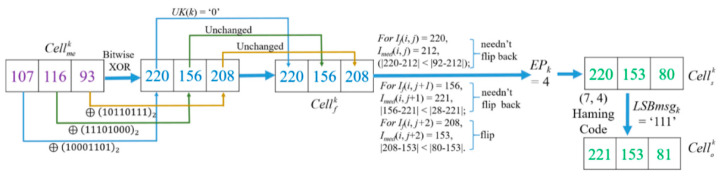
Example of image decryption procedure with *K_e_*.

*Example-5:* Follow the encrypted and hidden result demonstrated in Figure 13, its decryption procedure is shown in Figure 15. The flipping cases of *I_f_*(*i*, *j*) and *I_f_*(*i*, *j* + 1) are the same, they are determined as normal case because their pixel coordinates have not been recorded in EXCmsb. But being different from the former two pixels, the coordinate and MSB value of *I_f_*(*i*, *j* + 2) can be found in EXCmsb, and thus the recorded MSB ‘0’ is used to substitute the MSB of *I_f_*(*i*, *j* + 2) = 208 and form *I_s_*(*i*, *j* + 2) = 80. Then, *EP_k_* = 4 can be obtained according to the flipping rules listed in Table 1. In the meantime, on the basis of Figure 6, $CW^{\prime }$ = ‘1101000’ of $Cell_{k}$ is obtained. Based on the (7, 4) Hamming Code, by calculating Equation (4), the original *LSBmsg_k_* = ‘111’ is carried out. Finally, the original cell [221, 153, 81] is solved.

#### 3.7.3. Data Extraction and Image Decryption with Both Data Hiding and Encryption Key

With *K_e_* and *K_h_*, the receiver can not only recover the image but also extract the secret message.

*Step 1.* Generate the random {0, 1} bit stream using *K_e_*, then execute the bitwise XOR (EXCLUSIVE-OR) operation with the received marked encrypted image bit by bit. Thus, the receiver decrypts the marked encrypted image and gets ${I^{\prime }}_{e}$.

*Step 2.* Take the first l bits, bit by bit, out from the LSB bits of the marked encrypted image ${I^{\prime }}_{e}$ to form CAInfo. From the *l* + 1 position, take and save the LSB of ${I^{\prime }}_{e}$ to form the second part of the content of the scrambled secret message.

*Step 3.* Save the bits of the last two 3-bits units of USCs of Ime as they are the first part of the content of the scrambled secret message. Now all the scrambled cover message strings have been carried out. Then, by using *K_h_*, it is easy to recover the scrambled secret message and extract it completely with no errors.

*Step 4*. Recover the last two 3-bits units of USCs using the value of the first 3-bits unit in a cell. Now the flipped image *I_f_* is obtained.

*Step 5*. Flip back pixel values, pixel by pixel, according to two principles: (1) whether its coordinate has been recorded in EXCmsb and its corresponding MSB, (2) the comparison result of two difference values, one is the difference between the MED prediction values and the pixel value with original MSB, the other is the difference between the MED prediction value and the pixel value with flipped MSB.

*Step 6.* Extract the flipping information of MSBs in a cell to get the value of *EP_k_*. From *EP_k_*, the position where it is modified can be known, so that CW can be obtained. By substituting *X* value with $CW^{\prime }$ and calculated with Equation (2), the LSB value of the original image can be obtained. Hereto, the original image is completely recovered.

## 4. Experimental Results and Analysis

In our proposed HUD-RDHEI scheme, two kinds of vacated rooms can be created. The primary is derived from the LSBs of three continuous pixels. The reserved space will be filled by partial scrambled secrets by data hider with LSB substitution, which is different from [43,44,45]. As for the original LSBs of three continuous pixels, they will be embedded into non-LSB planes with (7, 4) Hamming Code for complete recovery later. Then, aiming to improve the hiding capacity, a novel UnitSmooth judging function called US was proposed to generate BMPus, which locates smooth units for carrying extra 6 bits of a secret message in a unit, thus a large hiding capacity can be realized.

**Figure 16:**
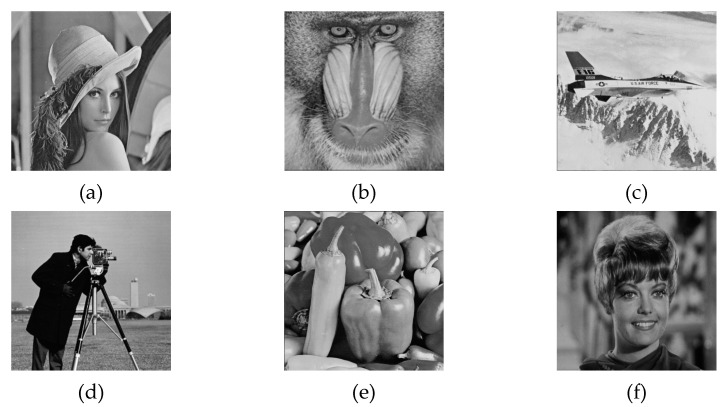
Test images (**a**) *Lena*, (**b**) *Baboon*, (**c**) *Airplane*, (**d**) *Cameraman*, (**e**) *Peppers*, (**f**) *Zelda*.

To further prove our performance on image quality and hiding capacity, sufficient the experimental results and comparisons of the proposed HUD-RDHEI scheme with several existing related works are demonstrated in this section. Some of the test images—Lena, Baboon, Airplane, Cameraman, Peppers, and Zelda—are downloaded from the Image Database SIPI [46]. They are shown in Figure 16. More experimental data is generated from two database images, BOSSBase [47] and BOWS-2 [48]. In the experiments, 20,000 grayscale images, with the size of 512 × 512, are used to evaluate the performance of the proposed HUD-RDHEI scheme. Here, the embedding rate (ER) is the measurement indicator to compare the proposed HUD-RDHEI scheme and some state-of-the-art algorithms. Peak signal-to-noise ratio (PSNR), correlation coefficient, number of pixels change rate (NPCR), and unified average changing intensity (UACI) are used to evaluate the reversibility of the proposed HUD-RDHEI scheme. Feature comparison of closely related methods are available and time complexity analysis is given.

Section 4.1 demonstrates the performance of the proposed HUD-RDHEI scheme. Section 4.2 presents the security analysis of the proposed HUD-RDHEI scheme. Finally, Section 4.3 displays the comparison results of the proposed HUD-RDHEI scheme and four existing schemes described in the Introduction, namely Puteaux et al.’s EPE-HCRDH [34], Puyang et al.’s Two-MSB-RDHEI [35], Yi et al.’s PBTl-RDHEI [36] and Chen et al.’s [37].

### 4.1. Performance Analysis of the Proposed HUD-RDHEI Scheme

As we mentioned in Section 3, the total embedding capacity of an image mainly depends on the quantity of UnitSmooth pixels and LSB, which minus CAInfo can obtain the pure payload. For a smooth image, the pixels satisfying the UnitSmooth condition account for a large proportion of all pixels in the whole image, which means a large amount of embedding capacity can be achieved. Conversely, for a complex image, such pixels are in a smaller proportion of all the pixels in the whole image, thus having less embedding capacity.

Table 3 shows the proportions of UnitSmooth pixels for the six test images, and the number of bits of CAInfo they require. The number of embeddable bits of a cell with UnitSmooth feature is 6, which are the 7th to 5th of the last two pixels of the cell. For an image, total embedding bits are the number of LSBs plus the number of USCs multiplied by 6. That can be calculated with Equation (9). Here NumOfLSB indicates the amount of LSBs except pixels located in the first row and first column; while NumOfUSC is the amount of units, of which the US value equals ‘1’ according to the calculation of Equation (1). Because the component of a cell is three pixels, the number of cells is 86,870:

(9) payload = NumOfLSB×1+NumOfUSC×6

(10) SizeOf(auxiliary information)= SizeOf(EXCmsb)×19 + SizeOf(BMPus)×1

The 1st column in Table 3 is the name of each test image. Value of the 2nd column tells the number of pixels whose coordinates and MSB values need to be hidden into the LSBs. For smooth images, this value is small. For example, ‘*zelda*’ and ‘*camera*’ have 0 and 43 pixels and are determined as abnormal cases, respectively. For complex images, for example, ‘Baboon’ has 4032 pixels determined as an abnormal case. It takes 12 bits to save a binary stream of ‘4032’ so that the receiver knows where the starting point of the second data set is. Moreover, two 4-bits are required; the first four bits indicate how many bits will be used to present the number of abnormal pixels, and the second four bits are used to record the binary stream of 12. Finally, the value of the 3rd column indicates size information of EXCmsb as 20 (=4 + 4 + 12). The 4th column of Table 3 indicates the number of USCs of an image, where every 6 bits of the secret message can be embedded into. The size of auxiliary information is calculated with Equation (10), and the content is compressed with the JBIG2 algorithm in [42] later to form CAInfo. After that, the pure payload comes from the value of the 5th minus the 6th column; that is to say, payload minus the size of CAInfo. The embedding rate (ER), shown in the 7th column, is the result of the pure payload divided by 512 × 512 = 262,144, which is the total number of pixels of a grayscale image. Equation (10) indicates the size of auxiliary information in bits, which includes the coordinates and MSB values of those pixels in abnormal situations and the bits of BMPus. The former means the column and row information of those abnormal pixels discussed in Section 4.1. The reason for multiplying by 17 is because the size of test image is 512 × 512 and 1 bit for recording MSB. The size of BMPus, named as SizeOfBMPus, is 86,870 bits (=511×⌊512/3⌋), which is the greatest number of cells defined in Section 3.1.

**Figure 17:**
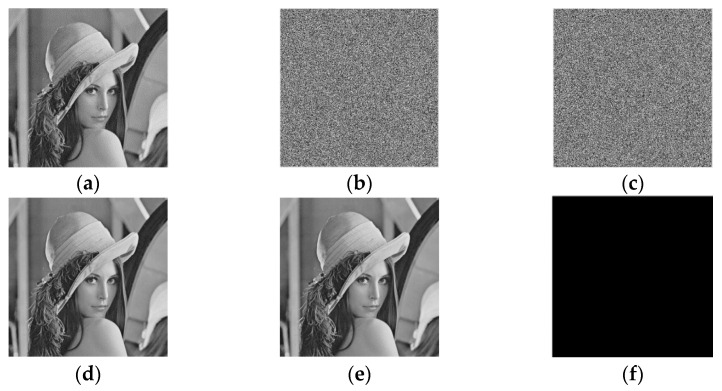
Results demonstration of the proposed HUD-RDHEII scheme (**a**) Original image; (**b**) Encrypted image; (**c**) Marked encrypted image with pure ER 2.389 bpp; (**d**) Directly decrypted image with PSNR +∞ and SSIM = 1; (**e**) Recovered image RSNR = +∞ and SSIM = 1; (**f**) The difference between (**a**) and (**e**).

Figure 17 shows the experimental results of applying the proposed HUD-RDHEI scheme to the ‘Lena*’* image. Due to the reversibility of the scheme, the original image can be restored completely and correctly, to be specific, directly decrypted image with PSNR +∞ and SSIM (structural similarity) = 1, recovered image RSNR = +∞ and SSIM = 1, which can be seen from Figure 17e,f. And the encrypted image and marked encrypted image, as shown in Figure 17b and Figure 17c, respectively, are random out-of-order images that the intruder cannot get effective information.

**Figure 18:**
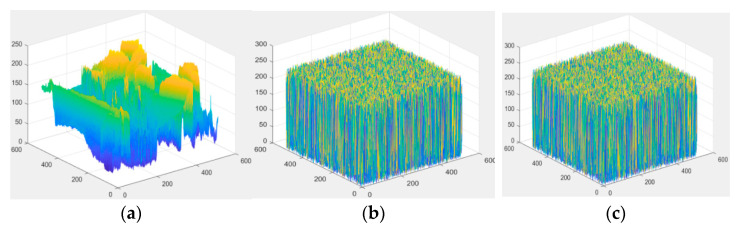
The 3D distribution of pixel values of ‘*Lena’* image. (**a**) The original image; (**b**) the encrypted image, and (**c**) the marked encrypted image.

To support this conclusion, the 3D distribution of pixels in these images is also listed in the following Figure 18. Figure 18 shows the pixel distribution of the original ‘Lena’ image, the encrypted ‘Lena’ image, and the marked encrypted ‘Lena’ image, respectively. From the result, we can see the pixel distribution of the encrypted image and the marked encrypted image is uniformly distributed. To a large extent, this can ensure that attackers cannot master the distribution and correlation between adjacent pixels. On the other hand, the confidentiality of image content in the cloud can be guaranteed, and the security of encrypted image secret information can be guaranteed. Therefore, this algorithm can achieve the goal of information hiding technology in the ciphertext domain, that is, it can not only protect digital images and prevent confidential information leakage, but also it can hide information, fully combining the advantages of image encryption and information hiding.

### 4.2. Security Analysis of the Proposed HUD-RDHEI Scheme

Table 4 shows PSNR value of each encrypted image and the correlation coefficient with its corresponding original image. It can be seen that after the encrypting process, each encrypted image has a PSNR of less than 10. So, it is difficult to realize the detection of the content of the original image *I_o_* from encrypted image *I_e_*. The correlation coefficient is a statistical that calculates the strength of the relationship between the relative movements of two variables, which is calculated as [49]. We use the correlation coefficient in Equation (11) to reflect the effect of the proposed HUD-RDHEI scheme:

(11) $$\rho =\frac{N\sum_{i=1}^{N}(x_{i}\times y_{i})-\sum_{i=1}^{N}x_{i}\times \sum_{i=1}^{N}y_{i}}{\sqrt{\left(N\sum_{i=1}^{N}x_{i}{}^{2}-({\sum_{i=1}^{N}x_{i})}^{2})\times (N\sum_{i=1}^{N}y_{i}{}^{2}-({\sum_{i=1}^{N}y_{i})}^{2}\right)}}.$$

Here *x* and *y* are gray values of two pixels with the same coordinates selected from *I_o_* and *I_e_*, and *N* is the total number of pairs of pixels of the image. The value ranges between −1.0 and 1.0. Once the calculation result value is greater than 1.0 or less than −1.0, conclusion can be got that an error exists in the correlation measurement. We can see that the correlation coefficients between the encrypted image and the original image are extremely close to zero, and so are the correlation coefficients between the marked encrypted image and the original images. It can be concluded that without the encryption key, it is impossible to obtain any valuable information from the encrypted image, nor from the marked encrypted image. For each 512 × 512 test grayscale image, as each bit of the image is encrypted at the bitwise level with a bit value generated by a random number, the encryption key space of the proposed HUD-RDHEI scheme is 2^512 × 512 × 8^, which is large enough to guarantee computing security. The encryption process of this scheme can protect and secure the privacy of the original image.

### 4.3. Comparisons with Closely Related State-of-the-Art Methods

**Figure 19:**
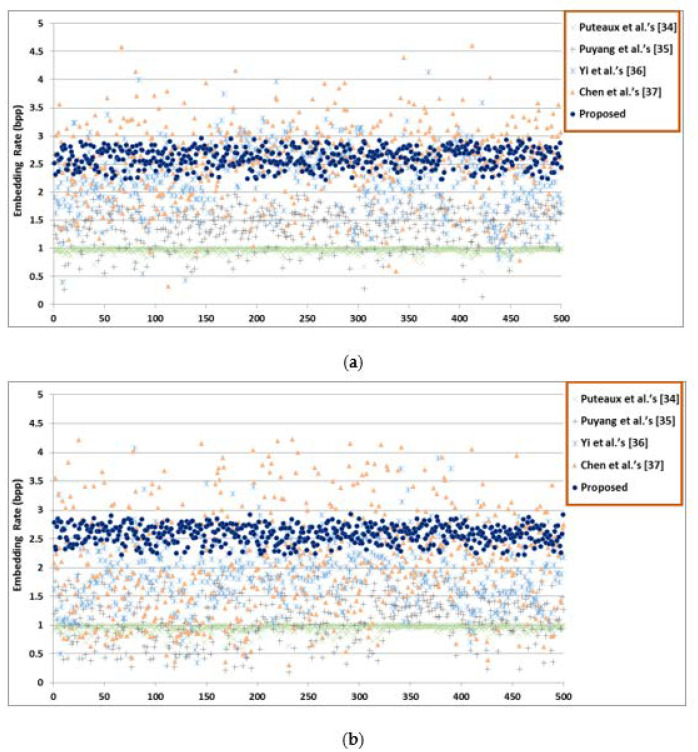
Average embedding rates of 500 marked encrypted images (**a**) images from BOSSBase; (**b**) images from BOWS-2.

In this subsection, first, the maximum and average ER of each method are compared in Table 5 and Table 6, then average embedding rates of 500 marked encrypted images from BOSSBase and BOWS-2 are shown in Figure 19. Next, feature comparison of the proposed HUD-RDHEI method with other state-of-the-art methods is provided in Table 7.

Table 5 shows the maximum ER comparisons of different images using the proposed HUD-RDHEI scheme and some state-of-the-art methods. Table 6 shows the average ER comparisons of different images based on 20,000 images from BOSSBase and BOWS-2 using the proposed HUD-RDHEI scheme and some state-of-the-art methods. The results show that the ER of our proposed algorithm is greatly improved than closely related state-of-the-art methods. In order to intuitively and visually see the difference of the ER distribution between the proposed HUD-RDHEI scheme and other algorithms, we randomly chose 500 images from two image datasets and generate the ER value of each image with five algorithms using a scatter diagram, as shown in Figure 19. It can be seen that the ER value of the scheme proposed by this algorithm is uniformly distributed, which is generally higher than other algorithms, and most of the values are very close to 3, regardless of if they are smooth or complex images. Although the algorithm from Yi et al.’s PBTl-RDHEI [36], and Chen et al. [37] have a higher ER on some pixels, this is with a very low percentage. Especially when we use the images in these two databases, each database has 10,000 images, to demonstrate the superiority of our algorithm over the other four algorithms on ER, as shown in Table 6. In summary, this scheme is obviously better than the four latest algorithms in terms of ER.

As depicted in Table 7, ref. [34] has two methods, corresponding to different features. The CPE method has some errors in the process of image restoration and decryption, while the EPE method does not. The proposed method is the same as the methods [35,36,37] in terms of separability, extraction, recovery and decryption with no errors.

According to the previous analysis combined with the features analysis, the proposed scheme will continue the features of separability, error-free recovery, error-free extraction and error-free decryption, and have a higher hiding capacity in both the maximum and average cases.

### 4.4. Time Complexity Analysis

Time complexity, also called the computation complexity, is often used to estimate the efficiency of an algorithm. It can measure the running time of an algorithm. In HUD-RDHEI, most of the time was spent on four parts. First, scanning image pixel values to hide LSB into non-LSB bits with (7, 4) Hamming code, recording modified position EP_k_, and flipping MSB of a pixel when needed. Second, encrypting original image with the simplest XOR stream encryption operation. Third, scrambling secret message and fourth, decrypting encrypted image and sequentially reading auxiliary information during data extracting and recovery phase. It is noted, this execution time of the latter three parts is proportional to the size of the cover image and the size of the auxiliary information. For content owner, data hider and receiver, they will take participant in specific process of the image encryption, data embedding, data extraction and image recovery process as needed. For an image with size of *M × N*, secret information with size of *S*, and auxiliary with size of *A*, the execution time of these four processes are O(*M × N*), O(*M* × *N* + *S* + *A*), O(*M × N* + *S* + *A*), O(*M × N* + *A*), respectively.

Next, we experimentally show time costing of the proposed HUD-RDHEI scheme. The measure of the time complexity is carried out over the MatLab implementation by using the built-in time function in a workstation equipped with an Intel i7-5500U @ 2.40 GHz CPU and 8 GB RAM. Take ‘Lena’ for example, under the condition of its maximum hiding capacity of 2.389 bpp, on the content owner side, it needs 4.490 s to preprocess the image to vacate room and encrypts the image to prepare for subsequent manipulation on the image. On the data hider side, 0.323 s and 0.495 s are needed to scramble secrets and embed secrets, respectively; on the receiver side, 0.646 s and 3.043 s are need to data extraction and image recovery, respectively.

## 5. Conclusions and Future Work

In this paper we have proposed a new high-capacity reversible data hiding scheme based on the encrypted domain. Our proposed scheme applies the (7, 4) Hamming Code to self-embed the LSB plane into the other planes of the image first, then adopts a novel US function to find more room to hide secret messages, and thus to improve the hiding capacity greatly. Through the use of the data hiding key, the embedded data can be fully extracted. Through the use of the encryption key, the original image can be recovered exactly from the decrypted image. Experimental results confirm that the average embedding rate of our proposed HUD-RDHEI scheme can be up to 2.556 bpp and 2.530 bpp under BOSSBase and BOWS-2, respectively, while guaranteeing the security of the hidden data. Certainly, areas of potential improvements might exist over our proposed scheme. For example, the 4th bit of the last two pixels of each unit does not participate in the activity of hiding secrets in our scheme. Thus, future research should work on concealing secret data using such space without damaging image quality. Furthermore, to extend the application of our proposed scheme to on-line tele-diagnosis, effectively reducing the execution time is another issue in need of addressing in the future.

## Figures and Tables

**Table 1:** Flipping combination of $MSB_{k}^{i}$ (1:flipped; 0: unflipped, i∈[1, 3]) used to identify *EP_k_*.

*EP_k_*	$MSB_{k}^{1}$	$MSB_{k}^{2}$	$MSB_{k}^{3}$
0	0	0	0
1	1	0	0
2	0	1	0
3	1	1	0
4	0	0	1
5	1	0	1
6	0	1	1
7	1	1	1

**Table 2:** The comparison result between *D_o-med_* and *D_o-fmed_* for four example 512×512 size grayscale images.

Images	*D_o-med_* > *D_o-fmed_*	*D_o-med_* = *D_o-fmed_*	*D_o-med_* < *D_o-fmed_*
*Lena*	111	6	261,004
*Baboon*	3630	289	257,202
*Airplane*	65	3	261,053
*Barbara*	1333	163	259,625

**Table 3:** The auxiliary information and embedding rate of six test images.

Images	NumOf- Abnormal	SizeOf- EXCmsb (bits)	NumOf- USC	Payload (bits)	SizeOf- CAInfo (bits)	Pure Payload (bits)	ER (bpp)
*Lena*	118	14	60,832	625,091	888	624,203	2.389
*Baboon*	4032	20	39,560	497,459	1514	495,945	1.902
*Airplane*	76	14	67,178	663,167	881	662,286	2.538
*Cameraman*	43	13	70,884	685,403	876	684,527	2.619
*Peppers*	141	16	58,886	613,415	891	612,524	2.345
*Zelda*	0	5	63,291	639,845	869	638,976	2.475

**Table 4:** PSNR of the encrypted images, marked encrypted images and correlation coefficients with the corresponding original images.

Images	PSNR (Marked Encrypted Image)	Correlation Coefficient	PSNR (Encrypted Image)	Correlation Coefficient
*Lena*	9.2292	−0.0009	9.2317	−0.0008
*Baboon*	9.5265	−0.0001	9.5289	−0.0005
*Airplane*	8.0330	0.0025	8.0260	0.0040
*Camera*	8.4077	−0.0003	8.4130	0.0002
*Peppers*	8.8831	−0.0007	8.8798	−0.0008
*Zelda*	8.8809	−0.0020	8.8773	−0.0021

**Table 5:** Maximum ER (embedding rate) comparisons of different images using the proposed HUD-RDHEI scheme and some state-of-the-art algorithms.

Images	Puteaux et al.’s [34]	Puyang et al.’s [35]	Yi et al.’s [36]	Chen et al.’s [37]	Proposed
*Lena*	0.977	1.155	2.014	1.944	2.389
*Baboon*	0.983	1.285	2.457	2.338	2.538
*Airplane*	0.839	0.377	0.462	0.535	1.902
*Cameraman*	0.990	1.531	2.442	2.648	2.619
*Peppers*	0.976	1.214	2.147	1.871	2.345
*Zelda*	0.996	1.321	2.474	2.110	2.475

**Table 6:** Comparison of the average ER(bpp) of two datasets between the proposed HUD-RDHEI scheme and five state-of-the-art methods based on 20,000 images from BOSSBase and BOWS-2.

Database	Puteaux et al.’s [34]	Puyang et al.’s [35]	Yi et al.’s [36]	Chen et al.’s [37]	Proposed
BOSSBase	0.996	1.447	1.957	2.434	2.556
BOWS-2	0.968	1.346	1.881	2.262	2.530

**Table 7:** Feature comparison of closely related methods.

Methods	Features			
	Separable	Error in Data Extraction	Error in Image Recovery	Error in Image Decryption
Puteaux et al.’s CPE [34]	Yes	No	Yes	Yes
Puteaux et al.’s EPE [34]	Yes	No	No	No
Puyang et al.’s [35]	Yes	No	No	No
Yi et al.’s [36]	Yes	No	No	No
Chen et al.’s [37]	Yes	No	No	No
Proposed	Yes	No	No	No
